# TRPC7 regulates the electrophysiological functions of embryonic stem cell-derived cardiomyocytes

**DOI:** 10.1186/s13287-021-02308-7

**Published:** 2021-05-03

**Authors:** Xianji Liu, Rui Zhao, Qianqian Ding, Xiaoqiang Yao, Suk Ying Tsang

**Affiliations:** 1 School of Life Sciences, The Chinese University of Hong Kong, Hong Kong SAR, China; 2 School of Biomedical Sciences, The Chinese University of Hong Kong, Hong Kong SAR, China; 3 State Key Laboratory of Agrobiotechnology, The Chinese University of Hong Kong, Hong Kong SAR, China; 4 Key Laboratory for Regenerative Medicine, Ministry of Education, The Chinese University of Hong Kong, Hong Kong SAR, China; 5 Institute for Tissue Engineering and Regenerative Medicine, The Chinese University of Hong Kong, Hong Kong SAR, China

**Keywords:** Embryonic stem cells, Cardiomyocytes, Canonical transient receptor potential isoform 7 channel, Electrophysiology

## Abstract

**Background:**

Biological pacemakers consisting of pluripotent stem cell-derived cardiomyocytes are potentially useful for treating bradycardia. However, tachyarrhythmia caused by derived cardiomyocytes themselves is one of main barriers hampering their clinical translation. An in-depth understanding of the mechanisms underlying the spontaneous action potential (a.k.a. automaticity) might provide potential approaches to solve this problem. The aim of this project is to study the role of canonical transient receptor potential isoform 7 (TRPC7) channels in regulating the automaticity of embryonic stem cell-derived cardiomyocytes (ESC-CMs).

**Methods and results:**

By Western blotting, the expression of TRPC7 was found to be increased during the differentiation of mouse ESC-CMs (mESC-CMs). Adenovirus-mediated TRPC7 knockdown decreased while overexpression increased the frequency of Ca^2+^ transients (CaTs), local Ca^2+^ releases (LCRs), and action potentials (APs) as detected by confocal microscopy and whole-cell patch-clamping. TRPC7 was found to be positively associated with the activity of ryanodine receptor 2 (RyR2), sarco/endoplasmic reticulum Ca^2+^-ATPase (SERCA), and sodium-calcium exchanger (NCX) but not hyperpolarization-activated, cyclic nucleotide-gated channel (HCN), and inositol trisphosphate receptor (IP3R). Knockdown or overexpression of TRPC7 did not alter the expression of HCN4, Cav1.3, Cav3.1, Cav3.2, IP3R1, RyR2, and SERCA but positively regulated the phosphorylation of RyR2 at S2814 and phospholamban (PLN) at T17. Moreover, the positive regulation of APs by TRPC7 was Ca^2+^-dependent, as overexpression of N-terminus of TRPC7 (dominant negative of TRPC7) which diminished the Ca^2+^ permeability of TRPC7 decreased the AP frequency.

**Conclusions:**

TRPC7 regulates the automaticity of mESC-CMs through two mechanisms. On the one hand, TRPC7 positively regulates the intracellular Ca^2+^ clock through the regulation of activities of both RyR2 and SERCA; on the other hand, TRPC7 also positively regulates the membrane clock via its influence on NCX activity. Altogether, our study reveals that TRPC7 is a potential drug target to manipulate the action potential firing rate of pluripotent stem cell-derived cardiomyocyte-based biological pacemakers to prevent tachyarrhythmia, a condition that might be encountered after transplantation.

## Background

Electronic pacing is a prevalent therapy to treat patients with symptomatic bradycardia or high-grade atrioventricular block. The world-wide survey suggested that around a million electronic pacemakers were transplanted in 2009 [1], and this number kept rising due to the aging population and the increasing number of patients with heart diseases [2, 3]. Despite their proven efficacy, electronic pacemakers still have some drawbacks, including limited battery life, device infections, lack of responsiveness to the autonomic nerves, and high magnetic fields incompatibility [4]. Therefore, the demand for developing better treatments is always existing. Benefiting from the fast development in the field of regenerative medicine, biological pacemakers which consist of cardiomyocytes derived from pluripotent stem cells (PSCs) have brought patients a promising adjunct or alternative therapy. Although proof-of-concept studies have demonstrated the potential of biological pacemakers to restore the heart rhythm in animal experiments, several safety concerns are still existing and hampering their clinical translation [5]. One of such concerns is tachyarrhythmia caused by the transplantation of PSC-derived cardiomyocytes because of their enhanced automaticity [6].

A crucial mechanism underlying the automaticity is the spontaneous occurrence of depolarization in the phase 4 of action potentials (APs). This spontaneous depolarization is determined by the ensemble of ion channels and exchangers on the plasma membrane which is defined as the membrane clock and rhythmic Ca^2+^ released from the sarcoplasmic reticulum (SR) which is regarded as the intracellular Ca^2+^ clock [7–9]. Inward funny current (*I*_f_) carried by hyperpolarization-activated cyclic nucleotide-gated (HCN) channels contributes to the early diastolic depolarization (DD) [10, 11]. During the mid and late DD, Na^+^-Ca^2+^ exchanger (NCX) generates an increasing inward current which leads to the acceleration of the late DD [12–14]. On the other hand, intracellular local Ca^2+^ releases (LCRs; also known as Ca^2+^ sparks) mediated by ryanodine receptor 2 (RyR2) occur spontaneously during the mid and late phase 4 and tick the time of Ca^2+^ clock. Because of the spatial proximity between SR and plasma membrane, Ca^2+^ released from RyR2 is moved out of the cell by NCX and this generates an inward current to accelerate late DD [12, 15]. By this way, the membrane clock and Ca^2+^ clock couple to each other to manipulate the spontaneous DD.

Although determinant proteins implicated in the automaticity have been well elucidated, the process is highly complicated, and more importantly, to ensure both robustness and elasticity of the automaticity, redundant mechanisms may exist for the regulation. Canonical transient receptor potential isoform 7 (TRPC7) is a non-selective cation channel with a higher selectivity of divalent cations over monovalent cations [16]. TRPC7 has been demonstrated to be a receptor-operated Ca^2+^ channel (ROCC). Specifically, G protein-coupled receptors (GPCRs) locating on the plasma membrane sense the external stimuli such as hormones, neurotransmitters, and growth factors transduce the signal to activate phospholipase C (PLC) which hydrolyzes the phosphatidylinositol 4,5-bisphosphate (PIP2) into inositol 1,4,5-trisphosphate (IP3) and diacylglycerol (DAG). TRPC7 is then directly activated by DAG, mediating the Ca^2+^ influx [17, 18].

In adult rat ventricular myocytes, TRPC3/7 heterotetramers were found to mediate an inward depolarization current and trigger arrythmia under the stimulation of ATP/UTP which is released in early infarct [19]. In neonatal rat ventricular myocytes (NRVMs), overexpression of TRPC7 was shown to induce apoptosis without/with angiotensin II stimulation probably through the calcineurin-dependent pathway, which may underlie the hypertension-induced heart failure [20]. Although these studies implied the potential functions of TRPC7 in the pathophysiology of cardiac diseases, the detailed mechanisms through which TRPC7 leads to the diseases are still elusive, mainly due to the absence of specific pharmacological agonists/antagonists and inhibitory antibodies of this channel. Moreover, both studies focused on functions of TRPC7 under pathological conditions, leaving its potential impacts on automaticity under normal physiological conditions unexplored. In light of the fact that TRPC7 relatives, including TRPM4 [21, 22], TRPM7 [23], and TRPC3 [24, 25], have been demonstrated to modulate the automaticity of cardiomyocytes, the aim of the present study was to investigate the role of TRPC7 in regulating the automaticity of developing cardiomyocytes using mouse embryonic stem cell-derived cardiomyocytes (mESC-CMs) which have been shown to generate sinoatrial nodal-like spontaneous APs [26, 27]. This study may help to further elaborate the mechanism underlying the automaticity of PSC-derived cardiomyocytes and provide some insights into potential solutions to conquer tachyarrhythmia caused by the transplantation of these cells.

## Materials and methods

### mESC culture and mESC-CM differentiation

mESCs from D3 line (ATCC, Manassas, VA, USA) were cultured as previously described [25, 28–30]. Briefly, mESCs were grown on Gamma-ray-irradiated mouse embryonic fibroblasts (MEFs), with Dulbecco’s modified Eagle’s medium (DMEM) (Invitrogen, Carlsbad, CA, USA) containing 15% heat-inactivated fetal bovine serum (FBS) (Hyclone, GE Healthcare, South Logan, UT, USA), 2 mM L-glutamine (Invitrogen), 0.1 mM non-essential amino acids (NEAA) (Invitrogen), 1% v/v penicillin-streptomycin (Invitrogen), 0.1 mM β-mercaptoethanol (Invitrogen), and 1000 U/mL leukemia inhibitory factor (LIF) (Chemicon, Millipore, Billerica, MA, USA) to maintain the undifferentiated status of mESCs. The medium was changed every day and the cells were passaged every other day. Differentiation was conducted through embryoid body (EB) method. Briefly, on day 0, cells were dissociated with 0.05% trypsin (Invitrogen) and resuspended in the medium without LIF. The cells were plated on a dish pre-coated with 0.1% gelatin (Sigma, St. Louis, Missouri, USA) and placed in the incubator for 30 min, allowing separation of mESCs from MEFs. mESCs suspending in the medium were collected and made into hanging drops, with each drop containing 20 μL medium and 800 mESCs. EBs were washed down to Petri dishes on day 2, maintained in suspension in 10 mL medium. On day 7, EBs were attached to dishes pre-coated with 0.1% gelatin and checked daily for the appearance of beating phenotypes.

### Isolation of mESC-CMs

Isolation of mESC-CMs was performed as previously described [25, 30]. Briefly, beating regions of EBs were dissected out and minced into small pieces with a 27-G needle under a dissection microscope. The EB pieces were centrifuged at 1000×*g* to remove medium and washed with pre-chilled PBS, then dissociated with 1 mg/mL collagenase B (Roche Diagnostics, Basel, Switzerland) in NB buffer containing 120 mM NaCl, 5.4 mM KCl, 5 mM MgSO_4_, 5 mM Na pyruvate, 20 mM glucose, 20 mM taurine, 10 mM HEPES, and 30 μMCaCl_2_, and pH was adjusted to 6.9 by NaOH. Dissociation was conducted at 37 °C for 20 min with gentle shaking. Collagenase B was then removed by centrifugation at 1000×*g* and 1 mL KB buffer was added to resuspend the cells. KB buffer was made of 85 mM KCl, 30 mM K_2_HPO_4_, 5 mM MgSO_4_, 1 mM EGTA, 5 mM pyruvic acid, 5 mM creatine, 20 mM taurine, 20 mM glucose, and 87 μM of Na-ATP (Sigma), and pH was adjusted to 7.2 by NaOH. The cells were further dissociated by gentle pipetting. Thereafter, they were plated on glass coverslips or confocal dishes for further use.

### Isolation of neonatal rat ventricular myocytes (NRVMs)

This study was approved by the Animal Experimentation Ethics Committee, the Chinese University of Hong Kong (17-006-MIS) and conformed to Guide for the Care and Use of Laboratory Animals published by the US National Institutes of Health (NIH Publication No. 80-23, revised 2011). Neonatal male rat pups (1–2 days postnatal) were sacrificed and the hearts were cut out. Atria were removed and ventricles of were cut into small pieces and washed in pre-chilled PBS on ice to remove blood. Ventricles were digested using 0.5 mg/mL collagenase type II (17101015, Gibco, Thermo) with gentle agitation. The digestion was ceased by adding DF-20 medium containing DMEM/F12 medium (11320033, Gibco, Thermo) and 20% FBS (Gibco, Thermo). Dissociated NRVMs were centrifuged at 1500 rpm, 4 °C for 10 min. The supernatant was discarded, and the cells were resuspended into plating medium containing DMEM/F12 medium, 5% FBS, 10% horse serum (26050088, Gibco, Thermo), and 1% v/v penicillin-streptomycin. The cells were plated to dishes or glass slides for further experiments.

### Molecular cloning

Mouse TRPC7 cDNA [16] was kindly provided by Professor Yasuo Mori (Kyoto University). For constructing TRPC7 overexpression plasmid Blue-pAdTrack-CMV-TRPC7, restriction endonuclease sites KpnI and NotI were added to 5′- and 3′- end of mTRPC7 cDNA, respectively, by PCR and the cDNA was ligated into the adenoviral shuttle plasmid Blue-pAdTrack-CMV which was modified from pAdTrack-CMV plasmid (Addgene plasmid # 16405). For constructing Tag-TRPC7 overexpression plasmid Blue-pAdTrack-CMV-TagTRPC7 KpnI site plus 3× Flag was added to the 5′- end, 3× hemagglutinin (HA) plus NotI site was added to the 3′- end of mTRPC7 cDNA, the cDNA was then ligated into Blue-pAdTrack-CMV. For constructing TRPC7 knockdown plasmids pAdTrack-U6-shRNA458 and pAdTrack-U6-shRNA459, two sequences of shRNA, shRNA-458 (targeting mouse TRPC7) and shRNA-459 (targeting rat TRPC7), adopted from Genetic Perturbation Platform were synthesized and ligated into the adenoviral shuttle plasmid pAdTrack-U6 at AgeI and XhoI restriction sites. pAdTrack-U6 was modified from pAdTrack plasmid (Addgene plasmid # 16404). The plasmid pAdTrack-U6-shRNAluc harboring shRNA targeting luciferase (shRNA-luc) was constructed as a negative control [31]. Targeting sequences of these shRNAs were shRNA-458: 5′-GCCGAATCAAACTCGCCATTA-3′, shRNA-459: 5′-GCCAACATTGAGACTGAATTT-3′, shRNA-luc: 5′-CCTAAGGTTAAGTCGCCCTCG-3′.

### Adenovirus production and infection

Adenovirus was prepared using AdEasy Adenoviral Vector System Kit (Agilent Technologies, Santa Clare, CA, USA) according to the manufacturer’s protocol. Briefly, the resultant adenoviral shuttle plasmids with gene-of-interest were linearized by the digestion of PmeI. The linearized shuttle plasmids were co-transformed with adenoviral backbone plasmid into *E. coli* BJ5183 cells by electroporation. The bacteria were spread onto kanamycin-containing agarose plates to grow for 24 h. Candidate recombinants which were the smallest colonies on the plates were picked and shaken in 5 mL kanamycin-containing LB medium for 16 h. Plasmids were extracted, and PCR and diagnostic restriction digestion were used to screen for the positive recombinants. Validated plasmids were transformed into *E. coli* DH5α cells for a further amplification. Finally, the recombinant plasmids were digested with PacI and transfected into HEK-293-AD cells with Lipofectamine 2000 (Invitrogen). Ten days after transfection, the cells were collected to extract adenoviruses by repeated freeze-and-thaw cycles. The viruses were further amplified in HEK-293-AD cells to gain a higher titration. For infection, viruses at 1 multiplicity of infection (MOI) were used for a moderate overexpression, and viruses at 20 MOI were used for a full knockdown. mESC-CMs or NRVMs were infected with viruses for 6 h in normal medium. Assays for overexpression were conducted 2 days after infection, and assays for knockdown were conducted 4 or 8 days after infection.

### Confocal Ca^2+^ imaging

Isolated mESC-CMs on confocal dishes were stained with 1 μM rhod-2 AM (Invitrogen) for 15 min in medium, then washed with pre-warmed Tyrode’s solution containing 1 mM MgCl_2_, 1.8 mM CaCl_2_, 5.4 mM KCl, 10 mM glucose, 10 mM HEPES, and 140 mM NaCl, pH 7.2 (adjusted by NaOH). The cells were bathed in Tyrode’s solution. Leica SP8 confocal microscope equipped with 552 nm laser and × 63 oil immersion objective was used for Ca^2+^ imaging. For the Ca^2+^ transients (CaTs) recording, the XYT mode was adopted. Images with a size of 16 × 16 pixels were captured in a frequency of 50 Hz for 1 min for each beating mESC-CM. For the LCRs recording, a XT line-scanning mode was used. A line in a length of 512 pixels was used to scan each beating cell in a frequency of 600 Hz for 13 s. ImageJ (NIH, Bethesda, MD, USA) was used to analyze the signal from CaTs images, a customized Python script was developed to analyze the parameters of CaTs. SparkMaster (University of California, Davis, CA, USA) was used for the analysis of LCRs [32].

### Electrophysiology

Membrane potential and ionic current were measured with the ruptured whole-cell patch clamp using Axopatch 200B amplifier (Molecular Devices, Sunnyvale, CA, USA) and pCLAMP 10.4 software (Molecular Devices) as previously described [25]. Signals were digitized at 10 kHz and filtered at 2 kHz. Microelectrodes (1B150F-4, World Precision Instruments, Sarasota, FL, USA) with resistances of 3–6 MΩ were pulled from P-97 puller (Sutter Instrument, Novato, CA, USA). For the AP recording, cells were bathed in Tyrode’s solution. The pipette solution was composed of 50 mM KCl, 80 mM KAspartate, 1 mM MgCl_2_, 3 mM MgATP, 10 mM EGTA, and 10 mM HEPES, pH 7.4 (adjusted with KOH). For NCX current (*I*_NCX_) measurement, cells were bathed in the solution containing 140 mM NaCl, 1.8 mM CaCl_2_, 1.2 mM MgCl_2_, 1 μM nifedipine, 20 μM ouabain, 1 μM ryanodine, 10 μM zatebradine, 5.5 mM HEPES, and 11 mM glucose, pH 7.4 (adjusted with NaOH). The pipette solution was composed of 65 mM CsCl, 20 mM NaCl, 5 mM Na_2_ATP, 1.75 mM CaCl_2_, 4 mM MgCl_2_, 10 mM HEPES, 20 mM tetraethylammonium chloride, and 5 mM EGTA, pH 7.2 (adjusted with CsOH). The following ramp-voltage-clamp protocol was used to elicit the *I*_NCX_: the potential was held at − 40 mV, then gradually increased to + 50 mV, followed by a decline to − 100 mV, finally increased back to + 40 mV, with a ramp rate of 200 mV/500 ms. *I*_NCX_ was indicated by the repolarization limb (from − 50 mV to − 100 mV) of the protocol as documented previously [25, 33]. For the recording of *I*_f_, cells were bathed in Tyrode solution supplied with 2 mM BaCl_2_ and 2 mM MmCl_2_ to suppress K^+^ and Ca^2+^ current [34]. The pipette solution contained 10 mM NaCl, 50 mM KCl, 80 mM KOH, 1 mM MgCl_2_, 10 mM HEPES, and 3 mM MgATP, pH 7.2 (adjusted with KOH). Five millimoles CsCl was applied to the bath solution to inhibit *I*_f_.

### Western blotting

One hundred micrograms proteins were loaded to 7.5% polyacrylamide gel and transferred to 0.45 μm PVDF membranes (Millipore). Membranes were blocked with 5% (w:v) milk for 1 h at room temperature then incubated with primary antibodies at 4 °C overnight. The membranes were washed with TBST three times and incubated with secondary antibody for 1 h at room temperature. Finally, the membranes were developed with Clarity Western ECL Substrate (Bio-Rad) and pictures were taken by ChemiDoc Touch (Bio-Rad). Antibodies used were anti-TRPC7 1:500 (HPA031126, Sigma), anti-β-tubulin 1:1000 (15,115, Cell Signaling, Danvers, Massachusetts, USA), anti-HCN4 1:200 (APC-052, Alomone), anti-Cav1.3 1:100 (ACC-005, Alomone), anti-Cav3.1 1:200 (ACC-021, Alomone), anti-Cav3.2 1:200 (ACC-025, Alomone), anti-RyR2 1:1000 (MA3-916, Invitrogen), anti-SERCA 1:200 (sc-30110, Santa Cruz), anti-IP3R 1:500 (ACC-019, Alomone), anti-p(S2814)RyR2 1:5000 (A010-31, Badrilla), anti-phospholamban (PLN) 1:1000 (A010-14, Badrilla), anti-p(T17) PLN 1:5000 (A010-13, Badrilla), HRP-conjugated goat anti-rabbit secondary antibody 1:5000 (Dako, Zug, Switzerland), and HRP-conjugated goat anti-mouse secondary antibody 1:5000 (Dako).

### Drugs and chemical reagents

Ouabain was purchased from the International Laboratory USA (South San Francisco, CA, USA). 2-2-Aminoethoxydipheylborate (2-APB) and thapsigargin were purchased from Tocris (Bristol, UK). Nifedipine, ryanodine, zatebradine, and all chemical reagents used to setup buffer solutions were purchased from Sigma.

### Statistical analysis

Three or more biological repeats were performed for each experiment. Data were presented as mean ± SEM. Data between two groups were compared by unpaired Student’s *t*-test. Data between three or more groups were compared by one-way analysis of variance (ANOVA) followed by Bonferroni’s multiple comparison tests. *P <* 0.05 was considered to be statistically significant.

## Results

### The expression of TRPC7 increased during the differentiation of mESC-CMs

**Fig. 1:**
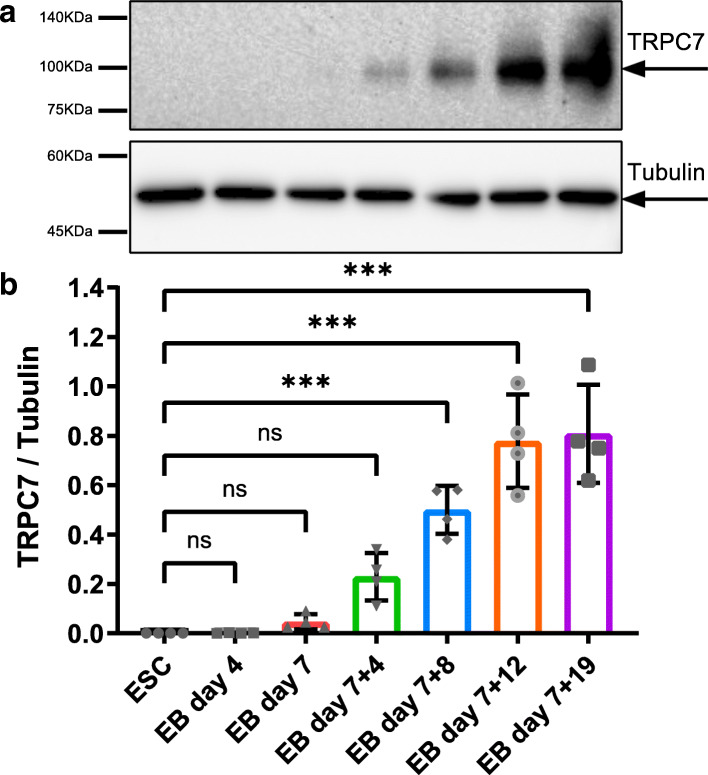
The expression of TRPC7 increased during the differentiation of mESC-CMs. **a** Representative Western blot showing the expression of TRPC7 at different stages. **b** Bar chart showing the quantification of TRPC7 normalized to β-tubulin. Data were presented as mean ± SEM (*n* = 4). ns, not significant, ***P < 0.001

To start our study, we first detected the expression of TRPC7 in mESCs at different stages during the differentiation. Cells were collected from mESCs and EBs on day 4, 7, 7 + 4, 7 + 8, 7 + 12, and 7 + 19, respectively; total proteins were extracted and subjected to Western blotting analysis. We found that neither mESCs nor EBs before their attachment to culture dishes (on day 7) expressed TRPC7. A significant increase of TRPC7 expression was observed since day 7 + 8 (8 days after attachment). TRPC7 expression kept rising until day 7 + 12 when it reached the plateau (Fig. 1). By co-staining of TRPC7 with cardiac markers, including cTnT and α-actinin, TRPC7 was also shown to be present in mESC-CMs and NRVMs (Supplementary Fig. 1).

### TRPC7 positively regulated the Ca^2+^ handling in mESC-CMs

**Fig. 2:**
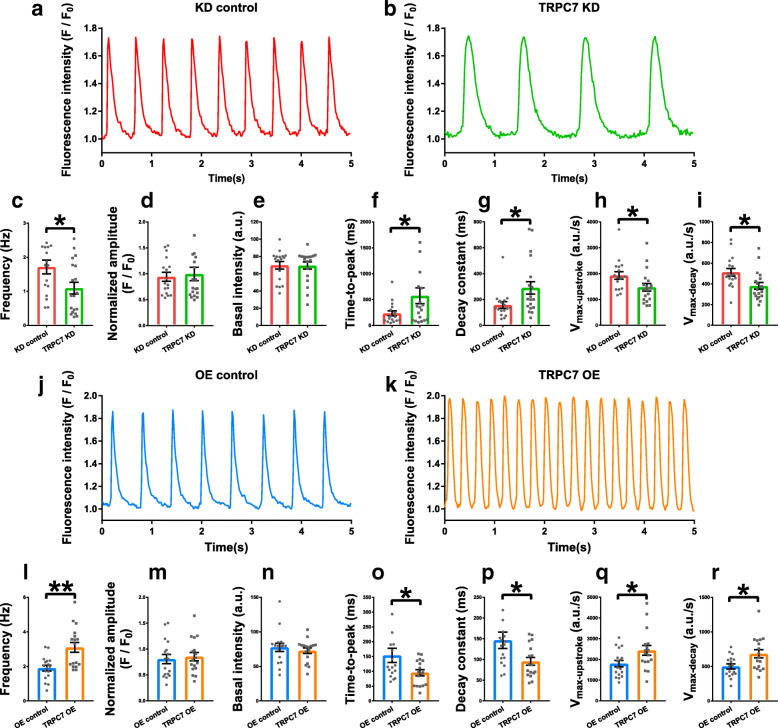
Knockdown of TRPC7 decreased while overexpression of TRPC7 increased the frequency of calcium transients (CaTs) in mESC-CMs. Representative CaTs of mESC-CMs recorded from **a** knockdown control (shRNA-luciferase) and **b** TRPC7 knockdown (shRNA-458) groups. Bar charts showing **c** the frequency, **d** the normalized amplitude, **e** the basal intensity, **f** the time-to-peak, **g** the decay constant, **h** the *V*_max-upstroke_, and **I** the *V*_max-decay_ of CaTs from **a** and **b**. Knockdown of TRPC7 decreased the frequency, *V*_max-upstroke_ and *V*_max-decay_, increased the time-to-peak and the decay constant and did not change the amplitude and the basal intensity of CaTs. Representative CaTs of mESC-CMs recorded from **J** overexpression control (BFP only) and **k** TRPC7 overexpression groups. Bar charts showing **l** the frequency, **m** the normalized amplitude, **n** the basal intensity, **o** the time-to-peak, **p** the decay constant, **q** the *V*_max-upstroke_, and **r** the *V*_max-decay_ of CaTs from **j** and **k**. Overexpression of TRPC7 increased the frequency, *V*_max-upstroke_, and *V*_max-decay_; decreased the time-to-peak and the decay constant; and did not change the amplitude and the basal intensity of CaTs. Data were presented as mean ± SEM (*n* = 17–19 cells; cells were from 3 independent batches of differentiation). *P < 0.05, **P < 0.01 vs corresponding controls

Ca^2+^ is important to various bioprocesses and cellular functions. In pacemaker cells, it is the foundation of Ca^2+^ clock which determines the automaticity. To study the function of TRPC7 in regulating the automaticity of mESC-CMs, Ca^2+^ handling was first investigated. Isolated mESC-CMs were infected with adenoviral vectors for knockdown or overexpression of TRPC7 (Supplementary Fig. 2). Our data showed that, in spontaneously beating mESC-CMs, compared with controls, knockdown of TRPC7 decreased (1.71 ± 0.20 vs 1.09 ± 0.18 Hz, *n* = 17–19, *P <* 0.05) (Fig. 2a–c), while overexpression of TRPC7 increased (1.90 ± 0.17 vs 3.10 ± 0.29 Hz, *n* = 17, *P <* 0.01) the frequency of CaTs (Fig. 2j–l).

The change of TRPC7 expression also led to an alteration of CaT kinetics. Specifically, when compared with controls, knockdown of TRPC7 increased the time-to-peak (233.71 ± 49.43 vs 574.79 ± 152.80 ms, *n* = 17–19, *P* < 0.05) and the decay constant (157.24 ± 26.10 vs 289.95 ± 48.09 ms, *n* = 17–19, *P <* 0.05) of CaTs, and decreased the maximum upstroke velocity (1928.48 ± 147.95 vs 1470.30 ± 143.69 a.u./s, *n* = 17–19, *P <* 0.05) and the maximum decay velocity (512.74 ± 36.51 vs 380.60 ± 32.41 a.u./s, *n* = 17–19, *P <* 0.05) (Fig. 2f–i), while overexpression of TRPC7 decreased the time-to-peak (153.82 ± 23.88 vs 95.29 ± 10.32 ms, *n* = 17, *P <* 0.05) and the decay constant (146.12 ± 19.82 vs 95.41 ± 9.61 ms, *n* = 17, *P <* 0.05) and increased the maximum upstroke velocity (1794.65 ± 158.36 vs 2432.61 ± 235.16 a.u./s, *n* = 17, *P <* 0.05) and the maximum decay velocity (499.02 ± 37.04 vs 682.73 ± 59.05 a.u./s, *n* = 17, *P <* 0.05) (Fig. 2o–r). On the other hand, neither knockdown nor overexpression of TRPC7 altered the amplitude and the basal level of CaTs (Fig. 2d, e, m, n). The data suggested that TRPC7 positively regulated the frequency and kinetics of CaTs.

**Fig. 3:**
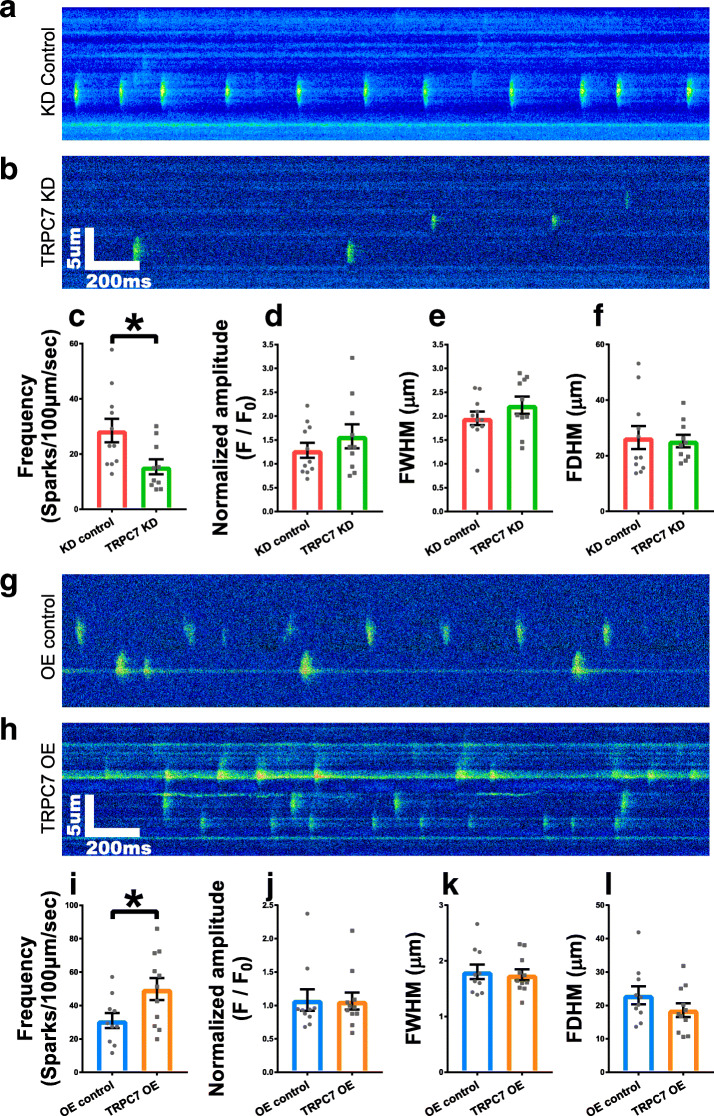
Knockdown of TRPC7 decreased while overexpression increased the frequency of localized calcium releases (LCRs) in mESC-CMs. Representative LCRs of mESC-CMs recorded from **a** knockdown control (shRNA-luciferase) and **b** TRPC7 knockdown (shRNA-458) groups. Bar charts showing **c** the frequency, **d** the amplitude, **e** the full width at half-maximum (FWHM), and **f** the full duration at half-maximum (FDHM) of LCRs from **a** and **b**. Knockdown of TRPC7 decreased the frequency of LCRs without affecting other parameters. Representative LCRs of mESC-CMs recorded from **g** overexpression control (BFP only) and **h** TRPC7 overexpression groups. Bar charts showing **i** the frequency, **j** the amplitude, **k** the FWHM, and **l** the FDHM of LCRs from **g** and **h**. Overexpression of TRPC7 increased the frequency of LCRs without affecting other parameters. Data were presented as mean ± SEM (*n* = 10–11 cells; cells were from 5 independent batches of differentiation). *P < 0.05 vs corresponding controls

Given the importance of LCRs in ticking Ca^2+^ clock and coupling SR Ca^2+^ release to the inward *I*_NCX_ to accelerate the late DD [7], whether TRPC7 would regulate LCRs was further tested. We found that, compared with controls, knockdown of TRPC7 decreased (28.52 ± 4.26 vs 15.39 ± 2.69 sparks/100 μm/s, *n* = 10–11, *P <* 0.05) (Fig. 3a–c), while overexpression of TRPC7 increased (30.97 ± 4.50 vs 49.84 ± 6.58 sparks/100 μm/s, *n* = 10–11, *P <* 0.05) (Fig. 3g–i) the frequency of LCRs. Neither knockdown nor overexpression of TRPC7 altered the amplitude, full width at half-maximum (FWHM), and full duration at half-maximum (FDHM) of LCRs (Fig. 3d–f, j–l). Our data suggested that TRPC7 positively regulated the frequency of LCRs without affecting the amplitude and kinetics of LCRs.

### TRPC7 positively regulated spontaneous APs in mESC-CMs by affecting the diastolic depolarization rate (DDR)

**Fig. 4:**
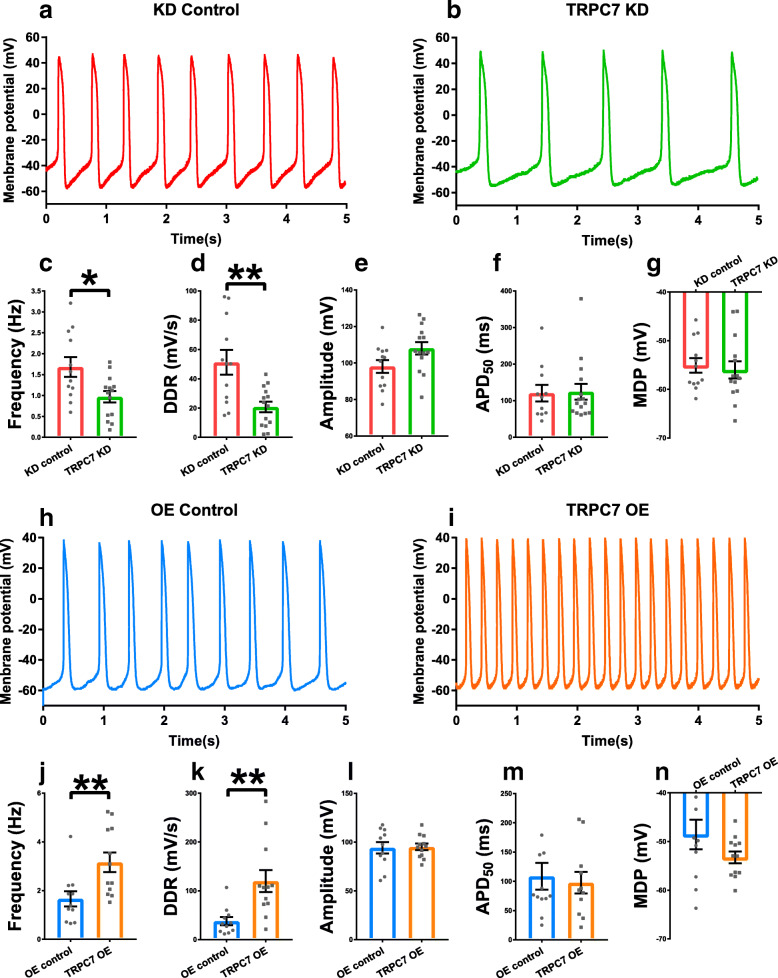
Knockdown of TRPC7 decreased while overexpression of TRPC7 increased the frequency of action potentials (APs) in mESC-CMs. Representative APs of mESC-CMs recorded from **a** knockdown control (shRNA-luciferase) and **b** TRPC7 knockdown (shRNA-458) groups. Bar charts showing **c** the frequency, **d** the diastolic depolarization rate (DDR), **e** the amplitude, **f** the 50% action potential duration (APD_50_), and **g** the maximum diastolic potential (MDP) of APs from **a** and **b**. Knockdown of TRPC7 decreased the frequency and DDR without affecting the amplitude, APD_50_ and MDP of APs. Representative APs of mESC-CMs recorded from **h** overexpression control (BFP only) and **i** TRPC7 overexpression groups. Bar charts showing **j** the frequency, **k** the DDR, **l** the amplitude, **m** APD_50_, and **n** the MDP of APs from **h** and **i**. Overexpression of TRPC7 increased the frequency and DDR without affecting the amplitude, APD_50_ and MDP of APs. Data were presented as mean ± SEM (*n* = 11–14 cells; cells were from 5 independent batches of differentiation). *P < 0.05, **P < 0.01 vs corresponding controls

Besides Ca^2+^ clock, membrane clock is another determinant of automaticity, we then moved on to examine if TRPC7 would regulate spontaneous APs. Whole-cell current-clamp technique was used to measure APs in spontaneously beating mESC-CMs. Consistent with the data of Ca^2+^ imaging, when compared with controls, knockdown of TRPC7 slowed down (1.68 ± 0.24 vs 0.97 ± 0.14 Hz, *n* = 12–14, *P <* 0.05) (Fig. 4a–c), while overexpression of TRPC7 accelerated (1.66 ± 0.26 vs 3.16 ± 0.40 Hz, *n* = 11–12, *P <* 0.01) (Fig. 4h–j) the AP firing rate in mESC-CMs. To further investigate how TRPC7 modulated the AP morphology and changed the rate of AP, several important parameters of AP were analyzed. The data showed that, compared with controls, knockdown of TRPC7 reduced (49.53 ± 8.43 vs 20.75 ± 3.61 mV/s, *n* = 12–14, *P <* 0.01) (Fig. 4d), while overexpression of TRPC7 increased (37.89 ± 8.35 vs 116.71 ± 22.42 mV/s, *n* = 11–12, *P <* 0.01) (Fig. 4k) the DDR without affecting other parameters including the amplitude, the APD_50_, and the maximum diastolic potential (MDP) of APs (Fig. 4e–g, l–n).

### TRPC7 positively regulated the activity of RyR2 and SERCA but not IP3R in mESC-CMs

**Fig. 5:**
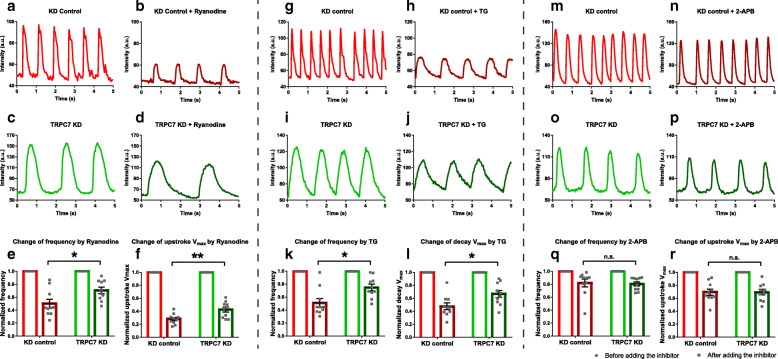
Knockdown of TRPC7 decreased the activity of RyR2 and SERCA without affecting the activity of IP3R in mESC-CMs. Representative CaTs recorded from the control group **a** before and **b** after applying ryanodine. Representative CaTs recorded from TRPC7 knockdown group **c** before and **d** after applying ryanodine. **e**, **f** Bar charts showing the normalized frequency and the *V*_max-upstroke_ calculated from **a**–**d**. The decrease of the frequency and the *V*_max-upstroke_ caused by ryanodine was less in TRPC7 knockdown group. Representative CaTs recorded from the control group **g** before and **h** after applying thapsigargin (TG). Representative CaTs recorded from TRPC7 knockdown group **i** before and **j** after applying TG. **k**, **l** Bar charts showing the normalized frequency and *V*_max-decay_ calculated from **g**–**j**. The decrease of the frequency and the *V*_max-decay_ and amplitude caused by TG was less in TRPC7 knockdown group. Representative CaTs recorded from the control group **m** before and **n** after applying 2-aminoethoxydipheylborate (2-APB). Representative CaTs recorded from TRPC7 knockdown group **o** before and **p** after applying 2-APB. **q**, **r** Bar charts showing the normalized frequency and the *V*_max-upstroke_ calculated from **m**–**p**. The decrease of the frequency and the *V*_max-upstroke_ caused by 2-APB was similar between control and TRPC7 knockdown groups. Data were presented as mean ± SEM (*n* = 10 cells; cells were from 3 independent batches of differentiation). *P < 0.05, **P < 0.01 vs control

Based on the observation that TRPC7 positively regulated the frequency and kinetics of CaTs, we sought to explore the underlying mechanism. To fulfill this purpose, TRPC7 was knocked down in mESC-CMs and the activity of RyR2, SERCA, and IP3R was subsequently evaluated as these proteins play critical roles in Ca^2+^ handling. A pharmacological method was adopted for the evaluation. Specifically, 10 μM ryanodine, 0.5 μM thapsigargin (Tg), and 20 μM 2-aminoethoxydipheylborate (2-APB) were applied to mESC-CMs to inhibit the activity of RyR2, SERCA, and IP3R, respectively; the response of cells was measured by Ca^2+^ imaging using confocal microscopy. We found that ryanodine decreased the frequency and the maximum upstroke velocity of CaTs in both control and TRPC7 knockdown mESC-CMs. But the extent of decrease was smaller in TRPC7-knockdown cells than control cells in terms of the frequency (50.15% ± 15.86% vs 70.49% ± 22.29%, *n* = 10, *P <* 0.05) and the maximum upstroke velocity (28.03% ± 2.54% vs 42.66% ± 3.86%, *n* = 10, *P <* 0.01) (Fig. 5a–f). Likewise, thapsigargin decreased the frequency and the maximum decay velocity of CaTs, with a smaller extent observed in TRPC7-knockdown cells than control cells in terms of both the frequency (51.44% ± 16.27% vs 75.14% ± 23.76%, *n* = 10, *P <* 0.05) and the maximum decay velocity (47.74% ± 5.24% vs 66.63% ± 5.39%, *n* = 10, *P <* 0.05) and (Fig. 5g–l). Although 2-APB decreased the maximum upstroke velocity and amplitude, the extent was similar between TRPC7-knockdown cells and control cells (Fig. 5m–r). We reasoned that knockdown of TRPC7 had already diminished the activity of both RyR2 and SERCA (but not IP3R), so smaller inhibitory efficacy was observed when blockers of RyR2 and SERCA (but not that of IP3R) were applied.

### TRPC7 positively regulated *I*_NCX_ but not *I*_f_ in mESC-CMs

**Fig. 6:**
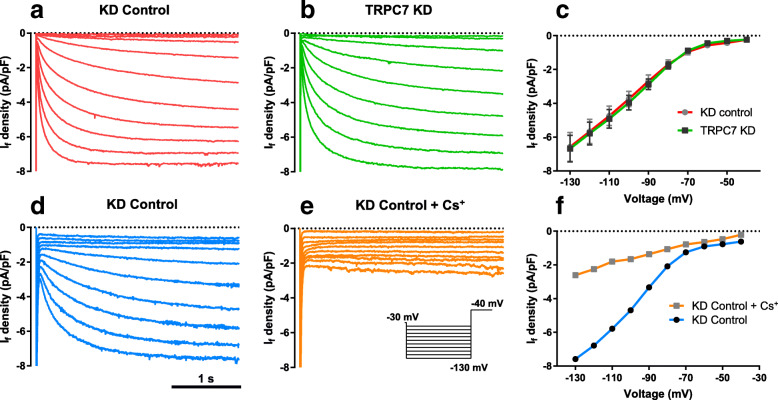
Knockdown of TRPC7 did not affect the activity of HCN in mESC-CMs. Representative *I*_HCN_ recorded from mESC-CM **a** knockdown control group and **b** TRPC7 knockdown group, respectively. **c** I-V curve of HCN calculated from **a** and **b**. TRPC7 knockdown did not change the amplitude of *I*_HCN._ Data were presented as mean ± SEM (*n* = 15 cells; cells were from 3 independent batches of differentiation). Representative *I*_HCN_ recorded from a mESC-CM knockdown control **d** before and **e** after adding Cs^+^, a specific blocker of HCN channels. **f** I-V curve of HCN calculated from **d** and **e**. The amplitude of current decreased after Cs^+^ application, indicating the current measured was *I*_HCN_

*I*_f_ has been regarded as the pacemaking current because it is the main contributor to the early DD. To study how TRPC7 positively regulates the frequency and DDR of APs, we knocked down TRPC7 in mESC-CMs and tried to detect the change of *I*_f_. We found that TRPC7 knockdown did not affect the *I*_f_ in mESC-CMs (Fig. 6a–c). To confirm the specificity of *I*_f_, 5 mM CsCl was applied to the bath solution of mESC-CMs infected with knockdown control adenovirus. It was found that CsCl dramatically decreased the current, confirming that the current record from this protocol was *I*_f_.

**Fig. 7:**
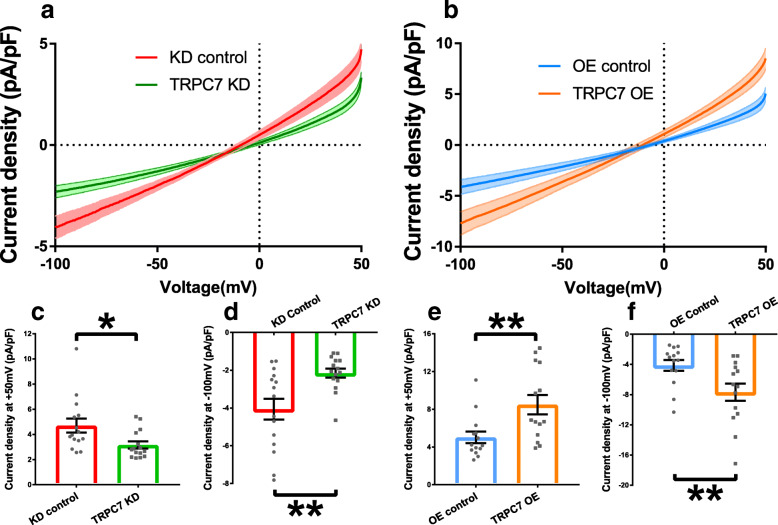
Knockdown of TRPC7 decreased while overexpression of TRPC7 increased the NCX current (*I*_NCX_) in mESC-CMs. **a** Current density-voltage relationship of *I*_NCX_ recorded from knockdown control mESC-CMs (red curve) and TRPC7-knockdown mESC-CMs (green curve). **b** Current density-voltage relationship of *I*_NCX_ recorded from overexpression control mESC-CMs (blue curve) and TRPC7-overexpression mESC-CMs (orange curve). Bar charts showing the statistics of NCX current density measured at + 50 mV and − 100 mV from **c**, **d**, the knockdown experiment, and **e**, **f** the overexpression experiment. Knockdown of TRPC7 decreased while overexpression of TRPC7 increased the outward *I*_NCX_ at + 50 mV and inward *I*_NCX_ at − 100 mV. Data were presented as mean ± SEM (*n* = 14–15 cells; cells were from 5 independent batches of differentiation). *P < 0.05, **P < 0.01 vs corresponding controls

In contrast to HCN channels which contribute to the early DD, NCX is believed to accelerate the late DD. We found that knockdown of TRPC7 decreased the *I*_NCX_ at both − 100 mV (− 4.06 ± 0.55 vs − 2.15 ± 0.24 pA/ pF, *n* = 15, *P <* 0.01) and + 50 mV (4.70 ± 0.56 vs 3.16 ± 0.28 pA/ pF, *n* = 15, *P <* 0.05) (Fig. 7a, c, d). On the other hand, overexpression of TRPC7 increased *I*_NCX_ at both − 100 mV (− 4.12 ± 0.71 vs − 7.68 ± 1.14 pA/ pF, *n* = 14, *P <* 0.05) and + 50 mV (5.02 ± 0.61 vs 8.48 ± 1.03 pA/ pF, *n* = 14, *P <* 0.01) (Fig. 7b, e, f). The data suggested TRPC7 could positively regulate the activity of NCX.

### TRPC7 did not affect the expression of several important proteins relevant to the automaticity

**Fig. 8:**
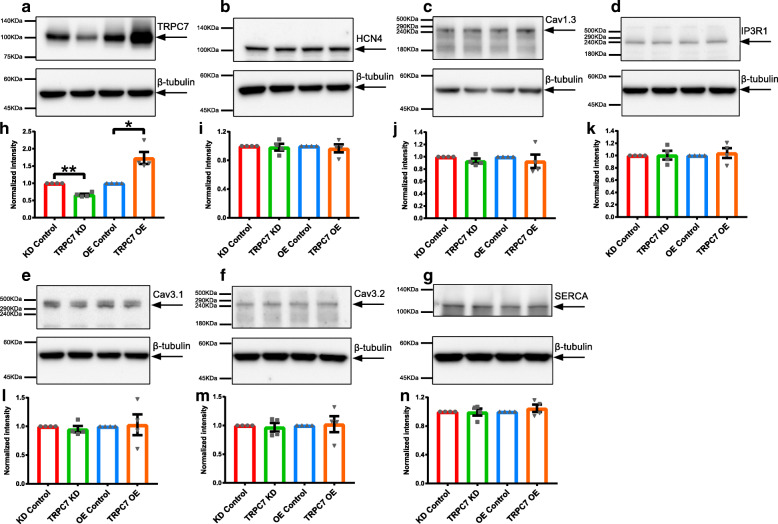
Knockdown or overexpression of TRPC7 did not alter the expression of several important ion channels/pump in NRVMs. **a**–**g** Western blots showing the expression of **a** TRPC7, **b** HCN4, **c** Cav1.3, **d** IP3R1, **e** Cav3.1, **f** Cav3.2, **g** SERCA in NRVMs infected with different adenoviruses to knockdown or overexpress TRPC7. **h**–**n** Bar charts showing the quantification of each protein from **a**–**g**. To eliminate the loading bias, intensity of each target protein was normalized to that of its corresponding β-tubulin. TRPC7 was successfully knocked down or overexpressed in NRVMs but the change of TRPC7 expression did not alter the expression of HCN4, Cav1.3, IP3R1, Cav3.1, Cav3.2, and SERCA. Data were presented as mean ± SEM (*n* = 4). *P < 0.05, **P < 0.01 vs corresponding controls

**Fig. 9:**
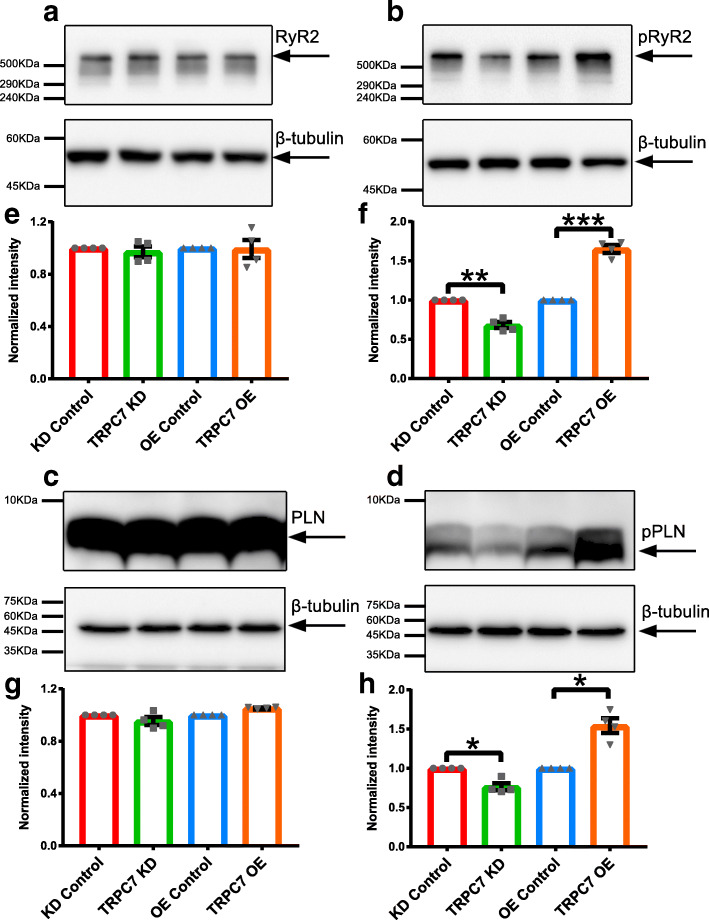
Knockdown of TRPC7 decreased while overexpression of TRPC7 increased the phosphorylation of RyR2 and PLN, respectively, in NRVMs. Western blotting showing the expression of **a** total RyR2, **b** p(S2814)RyR2, **c** total PLN, and **d** p(T17) PLN in NRVMs infected with different adenoviruses to knockdown or overexpress TRPC7. **e**–**h** Bar charts showing the quantification of each protein from **a**–**d**. To eliminate the loading bias, intensity of each target protein was normalized to that of its corresponding β-tubulin. The change of TRPC7 expression did not alter the expression of total RyR2 and PLN. However, knockdown of TRPC7 decreased while overexpression of TRPC7 increased the phosphorylation form of RyR2 [p(S2814)RyR2] and PLN [p(T17)PLN]. Data were presented as mean ± SEM (*n* = 4). *P < 0.05, ***P < 0.001 vs corresponding controls

TRPC7 is a Ca^2+^ permeable channel and Ca^2+^ entry is a very important mechanism by which gene expression pattern is modulated and cellular functions are shaped. Given the ability of TRPC7 to regulate the activity of the channel/pump/exchanger, we further examined whether TRPC7 could regulate the expression of several proteins relevant to the automaticity using NRVMs (which had a high percentage of purity; Supplementary Fig. 3). By Western blotting, the expression of HCN4, Cav1.3, Cav3.1, Cav3.2, RyR2, SERCA, and IP3R1 was detected after TRPC7 knockdown or overexpression in NRVMs. We found that, when compared to controls, neither knockdown nor overexpression of TRPC7 changed the expression of all these proteins (Figs. 8 and 9a, e).

### TRPC7 regulated the activity of RyR2 and SERCA through phosphorylation of RyR2 and PLN respectively

It has been well documented that phosphorylation of RyR2 and PLN regulated the activity of RyR2 [35] and SERCA [36], respectively. We had observed the positive regulation of TRPC7 to the activity of both RyR2 and SERCA. We then tested whether TRPC7 could affect the phosphorylation of RyR2 and PLN which in turn affect the activity of RyR2 and SERCA. TRPC7 was knocked down or overexpressed in NRVMs and phosphorylated RyR2 [p(S2814)RyR2] and phosphorylated PLN [p(T17)PLN] were detected by Western blotting. We found that while the total expression of RyR2 and PLN was not altered, both p(S2814)RyR2 and p(T17) PLN decreased after TRPC7 knockdown, and increased after TRPC7 overexpression (Fig. 9).

### Regulation of spontaneous APs by TRPC7 was Ca^2+^-dependent

**Fig. 10:**
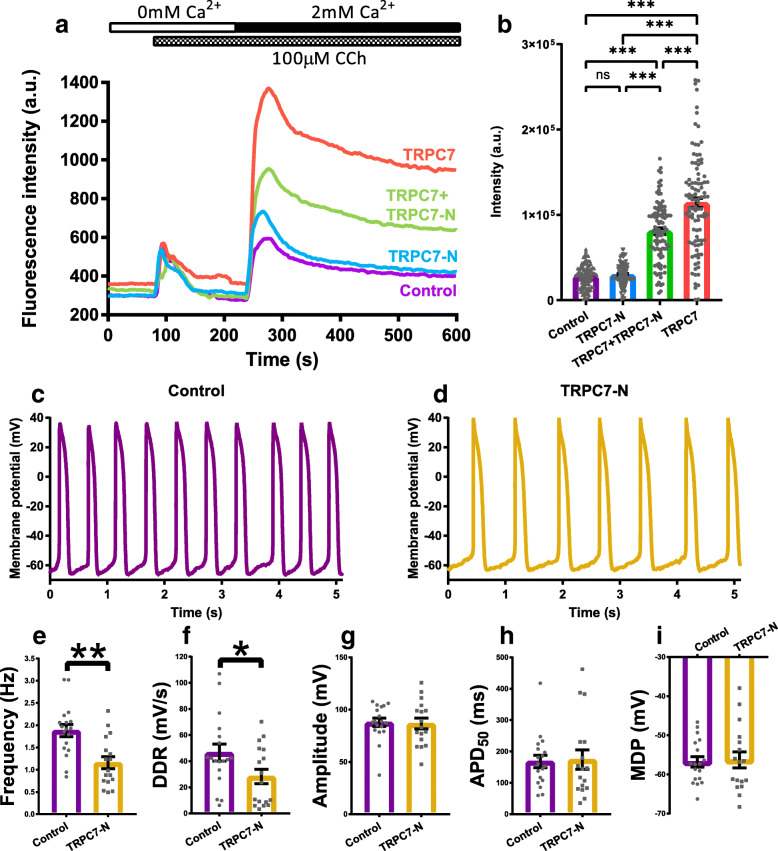
N-terminus of TRPC7 (TRPC7-N) partially diminished Ca^2+^ permeability of TRPC7 and decreased the frequency of AP in mESC-CMs. **a** Ca^2+^ imaging of HEK-293-FT cells transfected with different plasmids encoding TRPC7, TRPC7+TRPC7-N, and TRPC7-N respectively, empty plasmid was used as a negative control. Curves showed the average fluorescence intensity calculated from each group. The protocol used to elicit the Ca^2+^ entry via TRPC7 was shown on the top of the chart. **b** A bar chart showing accumulated intensity calculated from the area under each curve in **a** from 240 to 600 min. Co-expression of TRPC7-N with TRPC7 could decrease the Ca^2+^ entry mediated by TRPC7. Data were presented as mean ± SEM (*n* = 100 cells). ns, not significant; ***P < 0.001. Representative APs of mESC-CMs recorded from **c** overexpression control (BFP only) and **d** TRPC7-N overexpression groups. Bar charts showing **e** the frequency, **f** the DDR, **g** the amplitude, **h** APD_50_, and **I** the MDP of APs from **c** and **d**. Overexpression of TRPC7-N decreased the frequency and DDR without affecting the amplitude, APD_50_, and MDP of APs. Data were presented as mean ± SEM (*n* = 17–18 cells; cells were from 5 independent batches of differentiation). *P < 0.05, **P < 0.01 vs control

Due to the absence of specific pharmacological activators and inhibitors of TRPC7, it was difficult to directly dissect the effect exerted by the Ca^2+^ entry mediated by TRPC7. To overcome this problem, we developed a dominant negative of TRPC7, which consists of the N-terminus of TRPC7, to specifically diminish the Ca^2+^ permeability of TRPC7. The efficacy of TRPC7 dominant negative (TRPC7-N) was proven by the ectopic expression in HEK-293-FT cells and Ca^2+^ imaging. To measure the Ca^2+^ entry from the external environment, HEK-293-FT cells were initially bathed in Ca^2+^-free Tyrode’s solution. One hundred micromoles carbachol was applied to activate muscarinic acetylcholine receptors and subsequently PLC. Activation of PLC generated two products, IP3 and DAG which elicit Ca^2+^ release from ER (mediated by IP3R) and Ca^2+^ entry from the external environment (mediated by channels on plasma membrane), respectively. When the cells were bathed in Ca^2+^-free solution, the increase of intracellular Ca^2+^ represent the Ca^2+^ release from ER only. When the cytosolic Ca^2+^ was restored to a stable level, 1.8 mM Ca^2+^ was then applied to the bath solution, those channels activated by DAG now permeated Ca^2+^ and caused the cytosolic Ca^2+^ increase. By this method, Ca^2+^ entry from the external can be distinguished from Ca^2+^ release from ER. TRPC7-N was found not to mediate Ca^2+^ entry and co-expression of TRPC7-N with wild-type TRPC7 significantly decreased the permeability of TRPC7 (Figs. 10a–b). By overexpressing this TRPC7-N in mESC-CMs, we found that, when compared to the control group, APs (1.88 ± 0.14 vs 1.16 ± 0.13 Hz, *n* = 17–18, *P <* 0.01) and DDR (46.48 ± 6.51 vs 28.23 ± 5.49, *n* = 17–18, *P <* 0.05) were both decreased (Fig. 10c–i), similar to the effect of TRPC7 knockdown (cf. Fig. 4a–g). These results suggested that positive regulation of TRPC7 to the APs is Ca^2+^-dependent.

## Discussion

In this work, mESC-CMs were used as a model to study the function of TRPC7 in regulating the automaticity. The major findings are the following: (1) The expression of TRPC7 is increased during the differentiation of mESC-CMs. (2) TRPC7 can positively regulate the automaticity of mESC-CMs as shown by the positive regulation of CaTs, LCRs, and APs. (3) The regulation of CaTs by TRPC7 is implemented by regulating the activity of both RyR2 and SERCA but not their expression. (4) TRPC7 regulates the APs through the modulation of the NCX’s activity. (5) The effect of TRPC7 to the automaticity is Ca^2+^-dependent.

### TRPC7 positively regulates the Ca^2+^ clock of automaticity

Spontaneous, rhythmic Ca^2+^-driven events occurred on cardiac SR are referred to as the Ca^2+^ clock, a very important mechanism for pacemaker cells to realize the automaticity [8]. The functioning of Ca^2+^ clock requires LCRs (also called Ca^2+^ sparks) and CaTs. LCRs are spontaneous, and elementary Ca^2+^ release mediated by transient openings of clusters of RyR2 on SR [37]. LCRs usually occur at the late stage of DD before the upstroke of an AP. Studies have shown that even without the stimulation of APs, LCRs can still take place with a frequency similar to that of APs [38]. So LCRs are thought to be a time-keeper of the Ca^2+^ clock. CaTs are the results of the Ca^2+^ influx through voltage-gated L-type Ca^2+^ channel (LTCCs) (on the plasma membrane), and the bulk Ca^2+^ release from SR triggered by the Ca^2+^-induced-Ca^2+^ release mechanism. CaTs are regarded as a synchrony of considerable Ca^2+^ sparks [39, 40]; by this way, time of the Ca^2+^ clock is reset by APs every cycle. Our study showed that TRPC7 positively regulates both LCRs and CaTs and thus positively regulates the automaticity of mESC-CMs.

**Fig. 11:**
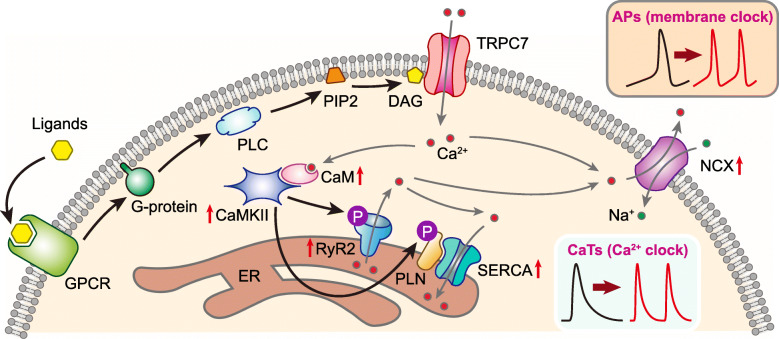
Schematic diagram illustrating the mechanism through which TRPC7 positively regulates the automaticity of cardiomyocytes. G protein-coupled receptors (GPCRs) locating on the plasma membrane (PM) sense the external ligands such as hormones, neurotransmitters, and growth factors, transduce the signal to activate phospholipase C (PLC) which hydrolyzes the phosphatidylinositol 4,5-bisphosphate (PIP_2_) into inositol trisphosphate (IP_3_) and diacylglycerol (DAG). TRPC7 is then directly activated by DAG, mediating the Ca^2+^ influx. Ca^2+^ permeated through TRPC7 may activate the forward mode of the Na^+^-Ca^2+^ exchanger (NCX), leading Na^+^ influx and depolarization. This depolarization would then accelerate diastolic depolarization (DD) and increase AP firing rate. On the other hand, Ca^2+^ permeated through TRPC7 may also increase the activity of ryanodine receptor 2 (RyR2) locating on the SR, probably through CaMKII and phosphorylation of RyR2, leading to an increase of the localized calcium releases (LCRs). These LCRs are then coupled to inward NCX current, and subsequently accelerate the DD and AP firing rate. At the same time, CaMKII may also increase the phosphorylation of PLN, which subsequently increases the activity of SERCA. The enhancement of the activity of both RyR2 and SERCA result in the acceleration of CaTs

RyR2 and SERCA are two most important proteins on the SR to keep the Ca^2+^ clock running properly. RyR2 contributes to the Ca^2+^ release from SR, which is the basis to generate LCRs and CaTs. On contrary to RyR2, SERCA is responsible for reloading SR with Ca^2+^ to ensure the continual function of the Ca^2+^ clock. It has been documented that phosphorylation of RyR2 and PLN (an inhibitory protein partner of SERCA when it is in an un-phosphorylated state) enhanced the activity of RyR2 [35] and SERCA [36], respectively, resulting in a positive regulation to the Ca^2+^ clock [41]. In line with previous reports, our data demonstrated that TRPC7 positively regulates the activity of RyR2 and SERCA via the phosphorylation of S2814 of RyR2 and T17 of PLN. We speculate that the phosphorylation may be mediated by CaMKII because S2814 of RyR2 and T17 of PLN are both catalytical sites of CaMKII [35, 36]. Since the activity of CaMKII is Ca^2+^-dependent, it is possible that TRPC7, via mediating Ca^2+^ entry, modulates the activity of CaMKII which positively regulates the phosphorylation of RyR2 and PLN and thereby the activity of RyR2 and SERCA (Fig. 11).

### TRPC7 positively regulates the membrane clock of automaticity

Various ion channels and exchangers located on the plasma membrane, which contribute to the spontaneous APs, work together to function as the membrane clock. Membrane clock is the other mechanism determining the automaticity. Our study showed that TRPC7 could positively regulate the spontaneous APs and therefore positively regulate the automaticity of mESC-CMs.

For those cardiac cells with automaticity (pacemaker cells), DD in phase 4 of APs is the most special feature to distinguish them from other working cardiomyocytes. After reaching the MDP in pacemaker cells, background current decreases while *I*_f_ current increases gradually, resulting in the early DD. While we found that TRPC7 positively regulates the DDR, TRPC7 exerts no impact on *I*_f_. The whole process of DD is like a relay race, while the athletes are different ion channels and an exchanger. The HCN channel kicks start the race, leading to the early DD, but *I*_f_ fades out near mid DD when the T-type Ca^2+^ channel becomes the next player, and NCX is the last player who contributes to the late DD. Our data demonstrated that TRPC7 positively regulates *I*_NCX_ and thereby positively regulates DDR.

It has been documented that the driving force of the activity of NCX mainly comes from Ca^2+^ released from SR though LCRs during late DD [7]. Because of the special proximity between SR and plasma membrane, Ca^2+^ released from SR could be moved out of the cells by NCX, thus generating a net inward current to further accelerate late DD. We speculate that TRPC7 positively regulates LCRs which subsequently regulates *I*_NCX_. But we cannot exclude the possibility that Ca^2+^ entry from TRPC7 could also directly activate NCX (Fig. 11).

## Conclusion

In conclusion, our study has demonstrated that TRPC7 is a positive regulator of the automaticity in mESC-CMs. On the one hand, TRPC7 positively regulates the intracellular Ca^2+^ clock through the regulation of activities of both RyR2 and SERCA; on the other hand, TRPC7 also positively regulates the membrane clock via its influence on NCX activity. This makes TRPC7 a potential drug target to manipulate the AP firing rate of PSC-CM-based biological pacemakers to prevent tachyarrhythmia, a condition that might be encountered after PSC-CM transplantation.

## Supplementary Information

**Additional file 1: Supplementary Figure 1.** TRPC7 was expressed in cardiomyocytes. A – B, Representative images showing immunostaining of nuclei (blue), TRPC7 (green) and (A) cTnT (red), (B) α-actinin (red) in mESC-CMs. C – D, Representative images showing immunostaining of nuclei (blue), TRPC7 (green) and (C) cTnT (red), (D) α-actinin (red) in NRVMs. The positive staining of cTnT and α-actinin suggested that these cells were cardiomyocytes, and the positive staining of TRPC7 indicated that the channel was expressed in cardiomyocytes. E, Representative images showing immunostaining of nuclei (blue), TRPC7 (green) and α-actinin (red) in NRVMs. In this experiment, the anti-TRPC7 was preincubated with its antigenic peptide to block its binding to TRPC7. The disappearance of TRPC7 staining signal suggested the specificity of the anti-TRPC7. Scale bars = 20 μm. **Supplementary Figure2.** TRPC7 was successfully knocked down and overexpressed in mESC-CMs. A – B, Overview of mESC-CMs infected by (A) TRPC7-knockdown adenovirus encoding GFP (Ad-CMV-GFP-U6-shTRPC7) and (B) TRPC7-overexpression adenovirus encoding BFP (Ad-CMV-TRPC7-CMV-BFP). Abundant GFP- and BFP-positive cells suggested that the infection efficiency of the adenoviruses was very high. C – D, Representative images showing immunostaining of α-actinin (yellow) and TRPC7 (red) in mESC-CMs infected by (C) knockdown-control and (D) TRPC7-knockdown adenoviruses. The positive signal of GFP (green) suggested that the cells were infected by adenoviruses. The intensity of TRPC7 signal decreased after infection with TRPC7-knockdown adenoviruses, suggesting the successful knockdown of TRPC7. E – F, Representative images showing immunostaining of HA (red) in mESC-CMs infected by (C) overexpression-control and (D) Tag-TRPC7-overexpression adenoviruses. The positive signal of BFP (blue) suggested that the cells were infected by adenoviruses. The intensity of HA signal increased after infection with Tag-TRPC7-overexpression adenoviruses, suggesting the successful overexpression of TRPC7. **Supplementary Figure 3**. The purity of cardiomyocyte was high in the NRVM preparation. A - B, Representative images showing immunostaining of nuclei (blue) and (A) cTnT (red), (B) α-actinin (red) in NRVM preparation. In the images, only two cells with an identity other than cardiomyocyte was found (indicated by arrows), while all others were cardiomyocytes (15 out of 17 were cardiomyocytes). The purity of cardiomyocyte estimated from these images was more than 85%. Scale bars = 20 μm.

## Data Availability

The datasets used and/or analyzed during the current study are available from the corresponding author on reasonable request.

## Abbreviations

2-APB: 2-Aminoethoxydipheylborate
APs: Action potentials
CaTs: Ca^2+^ transients
CMs: Cardiomyocytes
cTnT: Cardiac troponin T
DAG: Diacylglycerol
DD: Diastolic depolarization
EB: Embryoid body
ESC-CMs: Embryonic stem cell-derived cardiomyocytes
GPCRs: G protein-coupled receptors
HCN: Hyperpolarization-activated, cyclic nucleotide-gated channel
*I*_f_: Funny current
IP3: 1,4,5-Trisphosphate
IP3R: Inositol trisphosphate receptor
LCRs: Local Ca^2+^ releases
LTCCs: L-type Ca^2+^ channels
NCX: Sodium-calcium exchanger
NRVMs: Neonatal rat ventricular myocytes
PIP2: Phosphatidylinositol 4,5-bisphosphate
PLC: Phospholipase C
PLN: Phospholamban
PSCs: Pluripotent stem cells
ROCC: Receptor-operated Ca^2+^ channel
RyR2: Ryanodine receptor 2
SERCA: Sarco/endoplasmic reticulum Ca^2+^-ATPase
SR: Sarcoplasmic reticulum
TG: Thapsigargin
TRPC7: Canonical transient receptor potential isoform 7
