# Supplementary figures and images for: TRPC7 regulates the electrophysiological functions of embryonic stem cell-derived cardiomyocytes
Source: Stem Cell Res Ther. 2021 May 3;12:262. doi: 10.1186/s13287-021-02308-7 (PMC8091699; doi:10.1186/s13287-021-02308-7)

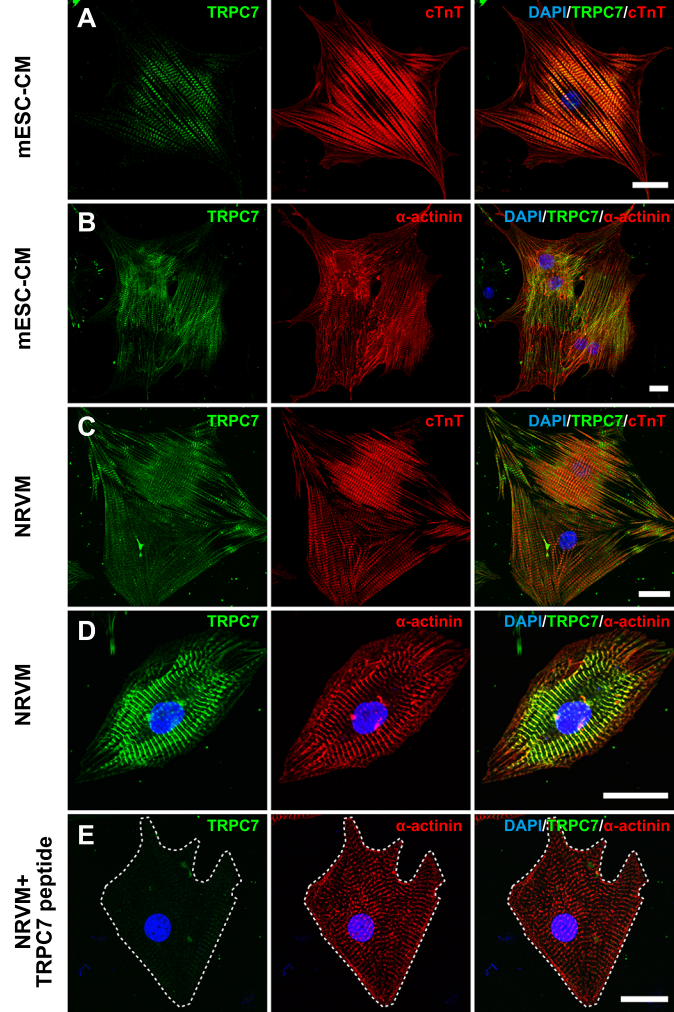

Supplementary figure 1

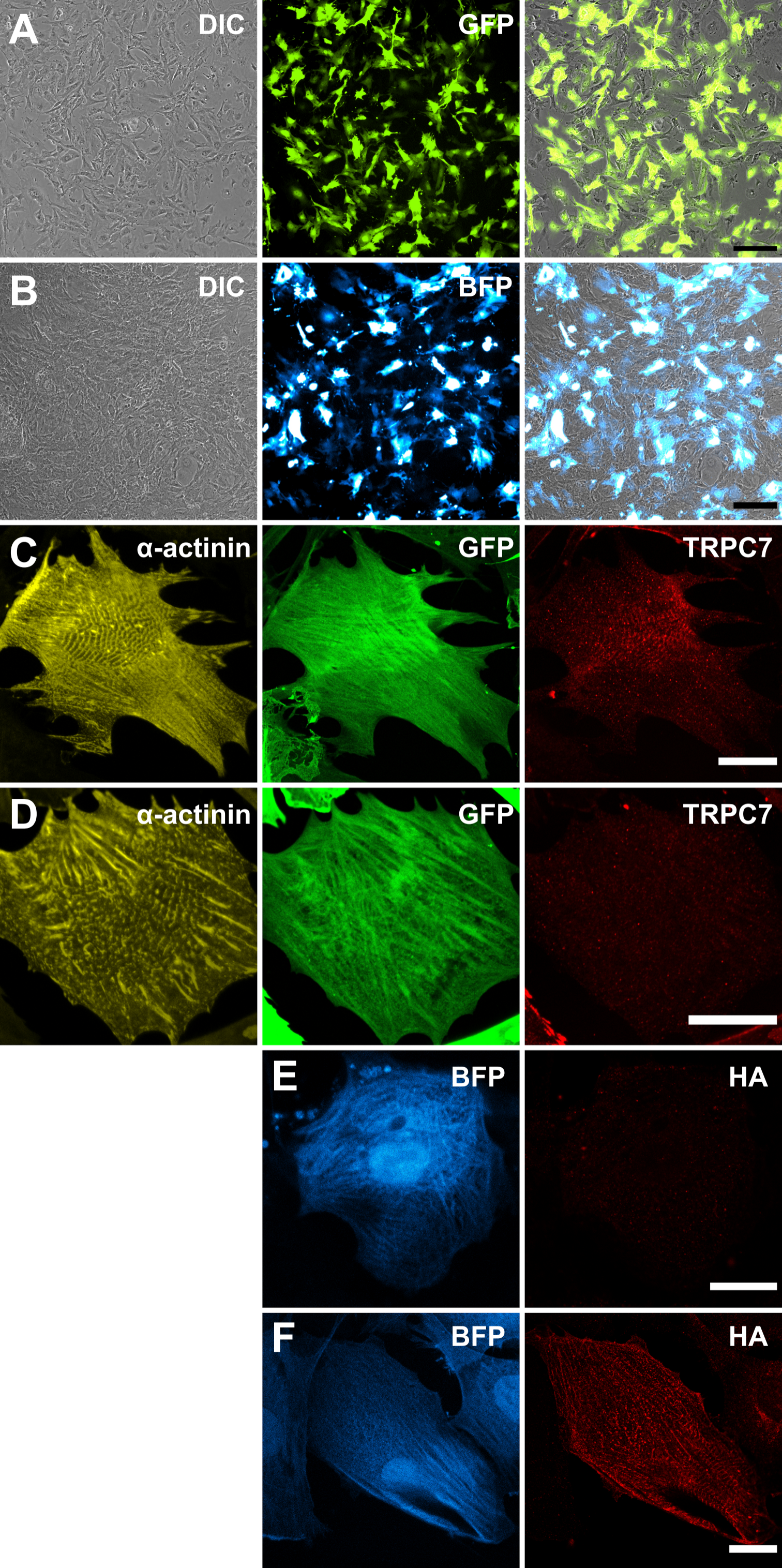

Supplementary figure 2

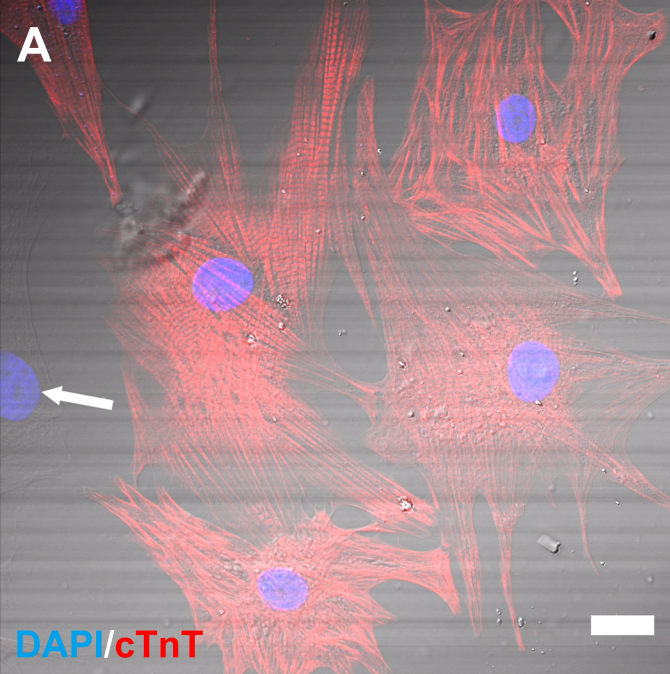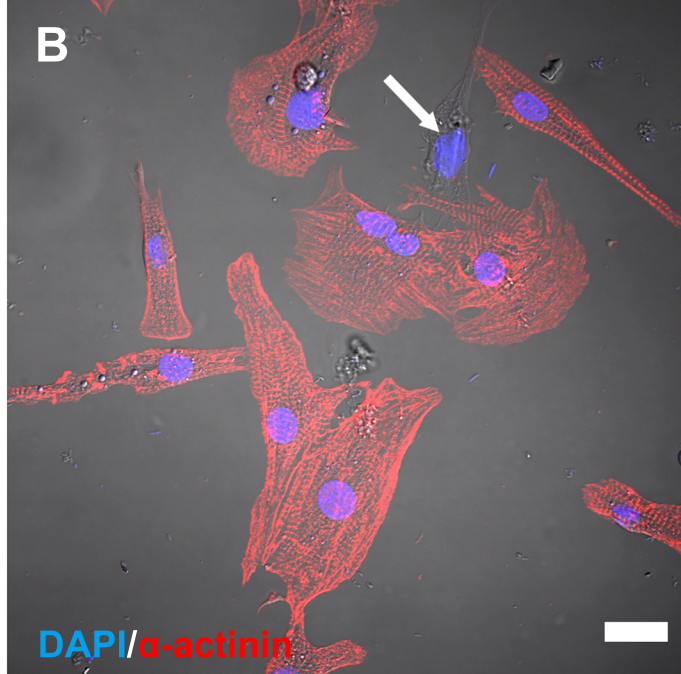

**Supplementary figure 3**

Supplement: Supplementary file 1 — Additional file 1: Supplementary Figure 1. TRPC7 was expressed in cardiomyocytes. A – B, Representative images showing immunostaining of nuclei (blue), TRPC7 (green) and (A) cTnT (red), (B) α-actinin (red) in mESC-CMs. C – D, Representative images showing immunostaining of nuclei (blue), TRPC7 (green) and (C) cTnT (red), (D) α-actinin (red) in NRVMs. The positive staining of cTnT and α-actinin suggested that these cells were cardiomyocytes, and the positive staining of TRPC7 indicated that the channel was expressed in cardiomyocytes. E, Representative images showing immunostaining of nuclei (blue), TRPC7 (green) and α-actinin (red) in NRVMs. In this experiment, the anti-TRPC7 was preincubated with its antigenic peptide to block its binding to TRPC7. The disappearance of TRPC7 staining signal suggested the specificity of the anti-TRPC7. Scale bars = 20 μm. Supplementary Figure2. TRPC7 was successfully knocked down and overexpressed in mESC-CMs. A – B, Overview of mESC-CMs infected by (A) TRPC7-knockdown adenovirus encoding GFP (Ad-CMV-GFP-U6-shTRPC7) and (B) TRPC7-overexpression adenovirus encoding BFP (Ad-CMV-TRPC7-CMV-BFP). Abundant GFP- and BFP-positive cells suggested that the infection efficiency of the adenoviruses was very high. C – D, Representative images showing immunostaining of α-actinin (yellow) and TRPC7 (red) in mESC-CMs infected by (C) knockdown-control and (D) TRPC7-knockdown adenoviruses. The positive signal of GFP (green) suggested that the cells were infected by adenoviruses. The intensity of TRPC7 signal decreased after infection with TRPC7-knockdown adenoviruses, suggesting the successful knockdown of TRPC7. E – F, Representative images showing immunostaining of HA (red) in mESC-CMs infected by (C) overexpression-control and (D) Tag-TRPC7-overexpression adenoviruses. The positive signal of BFP (blue) suggested that the cells were infected by adenoviruses. The intensity of HA signal increased after infection with Tag-TRPC7-overexpression adenovir [file 13287_2021_2308_MOESM1_ESM.pdf]
